# The Proposed Registration of Nurses

**Published:** 1889-12-21

**Authors:** 


### The Proposed Registration of Nurses.

*(Continued from page 148.)*

Metropolitan Hospitals, Nurse-Training Schools and Institutions Opposed to a Common Register, not Hitherto Published.

*London Hospitals.*

Accident Hospital, Poplar.—Emma Pilcher, matron.

Alexandra Hospital for Hip Disease.—Lucia Moore, lady superintendent.

Army Hospicals.—W. A. Mackinnon, Surgeon-General, Director-General Army Medical Staff.

British Lying-in Hospital.—E. J. Freeman, matron.

Cancer Hospital, Brompton.—Alex. Marsden, consulting and senior surgeon to the Cancer and Royal Free Hospitals; A. Rogers, matron.

Cheyne Hospital for Sick Children.—J. Prince Bartlett, senior surgeon; J. Macready, surgeon; E. N. Elam, lady superintendent.

Children's Hospital, Great Ormond-street.—Arthur Lucas, vice-chairman; Adrian Hope, secretary; K. Phillipa Hicks, matron.

City of London Hospital, Victoria Park.—K. S. Hetherington, matron.

City Orthopœdic Hospital.—Isley Pollard, matron.

East London Hospital for Children.—Charles Cheston, chairman; H. B. Donkin, M.B.Oxon, F.R.C.P.

Great Northern Central Hospital.—Mary Hall, matron.

Her Majesty's Hospital for Sick Children, Stepney.—S. A. Warburton, matron.

Homerton Hospital, Metropolitan Asylums Board.—Alex. Collie, M.D., medical superintendent; Emily Aston, matron.

Hospital for Consumption, Brompton.—William Beckwith, chairman; C. Theodore Williams, M.D., senior physician; Henry H. Taylor, F.R.C.S., resident medical officer and lecturer to nurses; Florence Abbott, lady superintendent.

Italian Hospital, Queen's-square.—W. Elgood, chairman.

Mildmay Nurses and Hospital, Stoke Newington.—James E. Mathieson, honorary superintendent and treasurer; J. F. Woodroffe, M.D., medical superintendent; Louisa Maxwell, matron.

Nursing Sisters' Institution, Devonshire-square, E.C.—F. M. Walrond and E. Still, members of committee; E. Rashdall, honorary secretary; A. B. Keeley, lady superintendent of nurses.

Middlesex Hospital.—A. Pearce Gould, M.S., F.R.C.S., assistant surgeon and lecturer to nurses.

Queen Charlotte's Hospital.—M. A. Phillips, matron and instructor of nurses.

Royal Chest Hospital, City-road.—M. L. Smith, matron.

Royal Free Hospital.—E. F. North, chairman; Samuel West, M.D., senior physician, assistant physician to St. Bartholomew's Hospital; A. Boyce Barrow, F.R.C.S., surgeon and lecturer to nursing staff.

Seamen's Hospital, Greenwich.—W. Johnson Smith, principal medical officer and lecturer to nurses; A. G. Cooke, matron.

Smallpox Hospital, Highgate.—S. Crockett, matron.

St. George's Hospital.—Hugh M. Macpherson, F.R.C.S., chairman of Nursing Committee; C. T. Dent, F.R.C.S., assistant surgeon and lecturer to nurses.

St. Pancras Infirmary.—Wm. Adams, F.R.C.S., J.P., chairman; C. Macann, medical superintendent; Ellen Jean Moir, matron.

Sydenham Hospital for Sick Children.—Edith Elmes, president; Georgiana Chapman, treasurer; E. Meadows, matron.

Temperance Hospital.—W. J. Collins, M.D., M.S., medical officer.

*District Nursing.*

Chelsea and Pimlico.—A. Hughes, superintendent.

East London Nursing Society.—Percy Wigram, treasurer; M. Curshaw Corner, medical officer; Louise Taylor, senior matron.

Metropolitan and National.—S. de Lüttichen, senior superintendent.

Paddington and Marylebone.—K. Persie, superintendent.

*Medical Homes.*

Beresford House, Portland-place.—Judith L. Stubbs, lady superintendent.

Devonshire-street, Portland-place, Nottingham-place, and Sevenoaks.—Catherine A. Veare, lady superintendent.

Gloucester-place, Portman-square.—Ellen Monk, lady superintendent.

Mansfield-street, Portland-place.—Bessie Boswell, lady superintendent.

Montague-place, Russell-square.—Bertha B. Moir, lady superintendent.

Upper Woburn-place.—Mary Cassal, lady superintendent.

Weymouth-street.—Mary Pollock, lady superintendent.

The Provincial and Scotch Nurse Training Schools will be given next week.

(*To be continued.*)

